# Is there a role for CDK 4/6 inhibitors in breast cancer brain metastases?

**DOI:** 10.18632/oncotarget.27904

**Published:** 2021-04-27

**Authors:** Ilana Schlam, Sara M. Tolaney

**Keywords:** breast cancer, brain metastasis, leptomeningeal disease, CDK 4/6 inhibitors

Breast cancer brain metastases (BCBM) continue to represent a challenge for clinicians as systemic treatment options remain limited and are associated with poor prognosis. Around 10–16% of patients with metastatic breast cancer will develop clinically significant central nervous system (CNS) involvement, including parenchymal or leptomeningeal disease (LMD) [1, 2]. The median survival of patients with parenchymal disease is 3 to 23 months and 3 to 4 months for those with LMD [3, 4]. The treatment of BCBM is based mostly on local therapies and there is an unmet need for systemic therapies for these patients, particularly for those with human epidermal growth factor receptor 2-positive (HER2+) tumors and for patients with LMD.

There are known differences in the incidence of brain metastases in specific breast cancer subtypes. Hormone receptor positive (HR+) is the most common breast cancer subtype [5]; however, the incidence of brain metastases in this subtype is the lowest (around 14%) [6]. HER2+ tumors account for 15% of breast cancers and the incidence of CNS involvement is highest in this subtype, up to 50% [5, 6]. About 10% of the patients with HER2+ breast cancer also have HR+ disease and around 35% of patients with HER2+/HR+ breast cancer will have CNS involvement [5, 6]. Triple-negative breast cancer accounts for 15% of breast cancer and around 25–46% of patients develop CNS disease [1, 7].

The cyclin dependent 4 and 6 (CDK4/6) inhibitors, palbociclib, ribociclib and abemaciclib, have changed the treatment paradigm of HR+ advanced breast cancer. Abemaciclib was initially approved by the United States Food and Drug Administration in 2017 in combination with endocrine therapy and as monotherapy. MONARCH 1 was a phase 2 study in which 132 women with heavily pretreated HR+/HER2- metastatic breast cancer received abemaciclib monotherapy (200 mg every 12 hours). The objective response rate (ORR) was 19.7%, with a median progression-free survival (PFS) of 6 months, and median overall survival (OS) of 17.7 months. Patients with brain metastases were excluded from this study [8]. MONARCH 2 was a phase 3 trial in which 669 women with HR+/HER2- metastatic breast cancer who progressed on endocrine therapy were randomized to receive abemaciclib (150 mg every 12 hours) with fulvestrant or placebo with fulvestrant. The addition of abemaciclib led to an improvement in the median PFS from 9.3 to 16.4 months (hazard ratio 0.55, 95% CI 0.449–0.681, *p* < 0.001) and also led to a significant improvement in OS (37.3 months vs 46.7 months, hazard ratio 0.75, 95% CI 0.606–0.945, *p* = .01). Patients with CNS disease were also excluded from this trial [9, 10]. Finally, MONARCH 3 demonstrated that among patients who were treatment naïve in the metastatic setting, adding abemaciclib to a nonsteroidal aromatase inhibitor significantly improved PFS (14.8 vs 28.2 months [hazard ratio 0.54, 95% CI 0.418–0.698, *p* = 0.000002]). Again, patients with breast cancer brain metastases were excluded from this study [11].

There is growing interest in expanding the use of targeted therapies, such as CDK4/6 inhibitors to patients with BCBM. Drug delivery into the CNS is a common barrier for the use of systemic therapy in brain metastases. Abemaciclib has shown blood brain barrier (BBB) penetration in animal models [12] and in a phase 1 study abemaciclib was detected in the cerebrospinal fluid (CSF) at similar concentrations of the ones unbound in plasma in patients with glioblastoma [13]. These findings confirmed CNS penetrance of abemaciclib, however, until recently the clinical efficacy in patients with breast cancer brain metastases was unknown.

A phase 2 trial was designed to evaluate the intracranial objective response rate (iORR) of patients with HR+ BCBM treated with abemaciclib [14]. Patients were enrolled into one of four cohorts: HR+/HER2- breast cancer; HR+/HER2+ breast cancer; LMD; and planned surgical resection of brain metastases. Patients received abemaciclib 200 mg twice daily or 150 mg twice daily in combination with trastuzumab, in selected patients with HER2+ disease. CNS penetrance of abemaciclib was confirmed in this study, as plasma and CNS concentrations were similar, consistent with preclinical studies. Moreover, among patients in the surgical resection arm who received abemaciclib prior to resection, the concentration of abemaciclib and its metabolites in CNS tumor tissue achieved levels expected to produce cell cycle arrest. Intracranial activity of abemaciclib was also demonstrated in this study. Of the 58 patients with HR+/HER2- disease, 3 patients had confirmed intracranial partial responses (iPR), resulting in confirmed iORR of 5.2%. The intracranial clinical benefit rate was 25% (95% CI 13.1–35.2), and the median intracranial PFS was 4.9 months. In the cohort of patients with HR+/HER2+ disease, there were no confirmed intracranial responses at the time of the interim analysis, so the study was stopped prior to proceeding to the second stage. There was evidence, however, of some clinical benefit, with 12 of the 27 patients experiencing intracranial stable disease (iSD), and among 3 patients, this lasted longer than 6 months. Additionally, in the LMD cohort, 7 patients with HR+/HER2- disease and 3 with HR+/HER2+ disease were enrolled, and one patient had a confirmed complete response. The median PFS for 5.9 months and the median OS was 8.4 months. These survival outcomes are longer than reported in historical series of patients with LMD [4, 14]. While this study did not meet its primary endpoint in terms of iORR, it confirmed that therapeutic doses of abemaciclib can be achieved in BCBM and that there is CNS activity of this medication. However, there still remain several unanswered questions.

One question is would abemaciclib have activity among patients with BCBM who have previously been treated with a CDK 4/6 inhibitor? Patients with metastatic HR+ disease often develop CNS metastases later in their disease course [6, 15], and CDK4/6 inhibitors are now standard of care first-line treatment for HR+/HER2- metastatic breast cancer, so patients will most likely be pretreated with a CDK 4/6 inhibitor at time of presentation of CNS metastases. While there are small case series that have shown systemic efficacy of abemaciclib after progression on palbociclib [16], we are awaiting data from randomized trials (NCT03147287, NCT02632045, NCT03809988) to know if there would be benefit with utilization of another CDK 4/6 inhibitor beyond progression. Patients with prior treatment with CDK4/6 inhibitors were not included in the study assessing the use of abemaciclib in BCBM [14]. Therefore, it is not clear if there is a role for this treatment in the setting of CNS disease at the time of progression on a different CDK4/6 inhibitor, and other combinations may need to be considered in the CDK 4/6 refractory setting to help overcome resistance.

Another question that remains is could there have been activity for abemaciclib for HR+/HER2+ BCBM if perhaps endocrine therapy and HER2-directed therapy with CNS activity had been utilized? Data from monarcHER suggested that among patients with HR+/HER2+ disease, adding endocrine therapy to abemaciclib and trastuzumab led to improved efficacy compared to chemotherapy and trastuzumab, whereas abemaciclib with trastuzumab without endocrine therapy performed very similarly to chemotherapy and trastuzumab [17]. These findings suggest that CDK 4/6 inhibitors may represent a good alternative to systemic chemotherapy and that endocrine therapy plays a significant role in the treatment of HR+/HER2+ disease [17]. Patients with untreated or progressive BCBM were not included in monarchHER. Additionally, there is now robust data for CNS efficacy of small tyrosine kinase inhibitors [18, 19]. In the HER2CLIMB study, the combination of tucatinib, capecitabine and trastuzumab led to an improvement in OS in patients with HER2+ BCBM when compared to capecitabine and trastuzumab (18.2 vs 12 months) [7]. Taken together, these data suggest that further work should be considered looking at abemaciclib with HER2 tyrosine kinase inhibitors and endocrine therapy for HR+/HER2+ BCBM.

Finally, a remaining question is could utilization of abemaciclib in the early disease setting help prevent CNS metastases? Data looking at adjuvant abemaciclib with endocrine therapy in monarchE among patients with high risk early stage HR+/HER2- breast cancer demonstrated that with a median follow up of 19.1 months there was an absolute benefit of 3% in invasive disease-free survival favoring the abemaciclib arm [20]. Longer follow up is needed to determine the long-term clinical benefit and effect on OS. While CNS events at time of first recurrence are not common among patients with HR+/HER2- breast cancer, with longer follow up we may be able to see if there are any signals that development of brain metastases can be prevented or delayed with adjuvant abemaciclib.

In summary, there are preclinical and now clinical data showing CNS penetrance and clinical activity of abemaciclib in patients with BCBM. Further studies are needed to explore the safety and efficacy of abemaciclib with other targeted agents, particularly for patients with HR+/HER2+ BCBM.
